# Production and characterization of polyhydroxyalkanoates by *Halomonas alkaliantarctica* utilizing dairy waste as feedstock

**DOI:** 10.1038/s41598-023-47489-8

**Published:** 2023-12-15

**Authors:** Justyna Mozejko-Ciesielska, Krzysztof Moraczewski, Sylwester Czaplicki, Vijai Singh

**Affiliations:** 1 Department of Microbiology and Mycology, Faculty of Biology and Biotechnology, University of Warmia and Mazury in Olsztyn, Oczapowskiego 1A, 10719 Olsztyn, Poland; 2 Institute of Materials Engineering, Kazimierz Wielki University, 85064 Bydgoszcz, Poland; 3 Department of Plant Food Chemistry and Processing, Faculty of Food Science, University of Warmia and Mazury in Olsztyn, Pl. Cieszyński 1, 10726 Olsztyn, Poland; 4 Department of Biosciences, School of Science, Indrashil University, Rajpur, Mehsana 382715 India

**Keywords:** Biotechnology, Biomaterials, Environmental biotechnology

## Abstract

Currently, the global demand for polyhydroxyalkanoates (PHAs) is significantly increasing. PHAs are produced by several bacteria that are an alternative source of synthetic polymers derived from petrochemical refineries. This study established a simple and more feasible process of PHA production by *Halomonas alkaliantarctica* using dairy waste as the only carbon source. The data confirmed that the analyzed halophile could metabolize cheese whey (CW) and cheese whey mother liquor (CWML) into biopolyesters. The highest yield of PHAs was 0.42 g/L in the cultivation supplemented with CWML. Furthermore, it was proved that PHA structure depended on the type of by-product from cheese manufacturing, its concentration, and the culture time. The results revealed that *H. alkaliantarctica* could produce P(3HB-co-3HV) copolymer in the cultivations with CW at 48 h and 72 h without adding of any precursors. Based on the data obtained from physicochemical and thermal analyses, the extracted copolymer was reported to have properties suitable for various applications. Overall, this study described a promising approach for valorizing of dairy waste as a future strategy of industrial waste management to produce high value microbial biopolymers.

## Introduction

Due to increasing environmental pollution, climate change, global warming and depleting petrochemical resources, bio-based platforms have gained significant attention and interest^1^. Synthetic polymers are widely used for several applications including bags, food packaging, containers, bottles, tubes and pharmaceuticals and cosmetics. However, nowadays, they are major pollution in terrestrial and aquatic environments^2^. In the past decade, biopolymers of microbial origin have attracted much attention as sustainable alternatives to conventional materials that cause a major environmental concern. They have many advantages over petrochemical-based polymers, including biodegradability, biocompatibility, non-toxicity and increased sustainability^3,4^. They are used for many applications, such as food packaging, medical implants, and biodegradable polymers^5^.

Microbial biopolyesters are known as polyhydroxyalkanoates (PHAs), an eco-friendly substitute for synthetic polymers derived from petrochemical refineries^6^. However, their industrial production is limited due to the overall costs of their production process compared to synthetic polymers^7^. There are many challenges for the microbial production of PHA such as microbial strains, raw materials, fermentation optimization and extraction. Many attempts have been made to optimize novel bioprocesses using low-cost carbon sources, especially those generated by the food industry.

Currently, the increasing demand for milk and its products is high globally. At the same time, dairy waste is also produced and released into wastewater, which causes serious issues to aquatic animal health. Approximately 11 million tones of dairy waste is released annually into the environment worldwide^8^. Therefore, there is a pressing need to use dairy waste and convert it into high-value chemicals for sustainable production. In particular, cheese production worldwide is gradually increasing and producing two major by-products: whey permeate and whey mother liquor. Whey permeate, also known as mother liquor, is a liquid separated from lactose crystals and used as a source for food grade lactose. The latter is not recovered and causes problems in waste management. Therefore, it could be considered a promising carbon source for microbial PHA production. There is a need to isolate and identify microorganisms that can utilize cheaper carbon sources and produce high amounts of PHAs.

Extremophiles are microorganisms living in extreme environments that would be fatal to most other life forms. Nevertheless, they are of great interest to scientists because of their insights into how life can adapt to extreme conditions. Furthermore, they also have practical applications in fields such as biotechnology, as some produce bioproducts that are useful in industrial processes^9^. Saline environments are widely distributed around the world. They are a source of halophilic bacteria that have a great potential to produce chemicals and biomolecules^10,11^.

Most recently, Hintersatz et al.^12^ isolated two siderophore-producing strains, *Halomonas gemina* sp. nov. and *Halomonas llamarensis* sp. nov. from hypersaline, alkaline surface waters of high-altitude salars of the Atacama Desert. It has been discovered that *Halomonas* spp. can produce PHAs, a biopolymer from renewable resources^13^. Some of these bacteria, such as *Halomonas nitroreducens*^14^, *Halomonas campisalis*^15^ or *Halomonas halophila*^16^ seem to be promising candidates for PHA production which are capable of utilizing a variety of cheaper carbon sources. However, *Halomonas* species have not been studied for PHA production on cheese whey mother liquor that is generated by the dairy industry.

Currently, no study has evaluated the potential of *Halomonas alkaliantarctica* for the production of PHAs, even though this species has substantial biotechnological potential in exopolysaccharide production^17^. Therefore, the aim of this study was to investigate the potential of this unexplored *H. alkaliantarctica* for the production of PHAs from bioproducts generated by cheese manufacturing. The study evaluated the impact of waste substrate concentration on biomass rate, PHA productivity, and monomeric structure and determined physico-thermal and water related properties.

## Results and discussion

### Influence of dairy industry waste on the growth of *Halomonas alkaliantarctica* and PHA production

PHA production costs are still high compared with petrochemical derived polymers. Bioprocesses that rely on halophilic production species are advantageous as they can result in reduced production costs^18^. Extremophiles are resistant to microbial invasion, which allows for continuous cultivation^19^. Additionally, PHAs can be extracted from bacterial cells through hypotonic cell lysis, which further decreases the cost of biopolymer extraction^20^. Halophilic bacteria have also been recently discovered to be capable of utilizing various waste feedstocks into high-value products^17^. Therefore, the study aimed to evaluate the potential of promising extremophiles for the biotechnological production of PHA from bioproducts generated during cheese manufacturing. There are no reports that evaluated the potential of *H. alkaliantarctica* to grow and produce PHAs in cultivations supplemented with CW and CWML. In addition, *Halomonas* spp. has not yet been investigated for these biopolymers production on CWML. Therefore, it was decided to study the effect of dairy waste in the cultures of *H. alkaliantarctica* on its growth rate and efficient PHA synthesis and accumulation.

**Figure 1:**
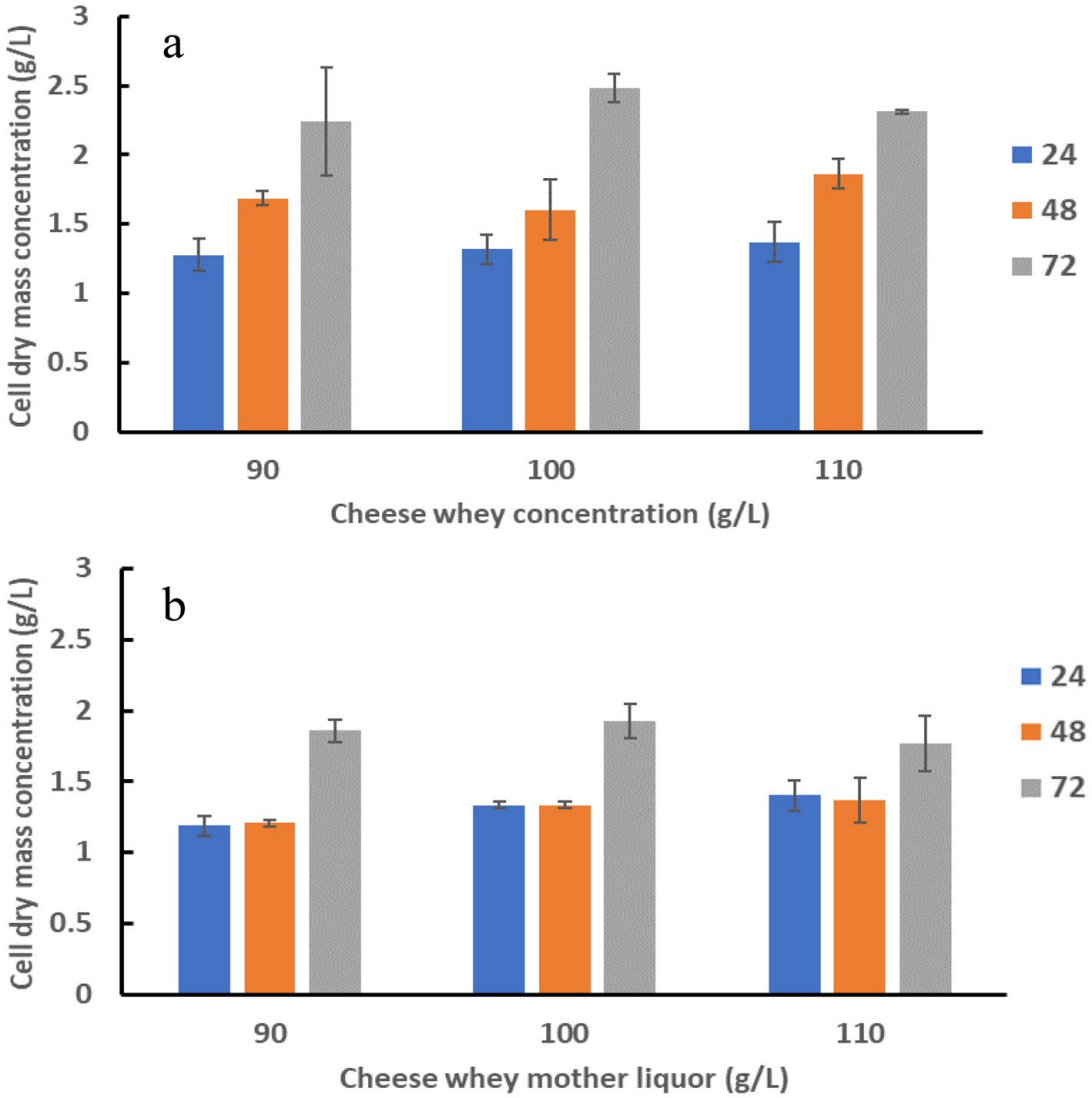
Cell dry mass concentration (g/L) in 24 h, 48 h and 72 h of the cultivations with: (**a**) cheese whey and (**b**) cheese whey mother liquor at the concentrations of 90, 100 and 110 mL/L. Mean values are calculated from triplicate measurements.

**Figure 2:**
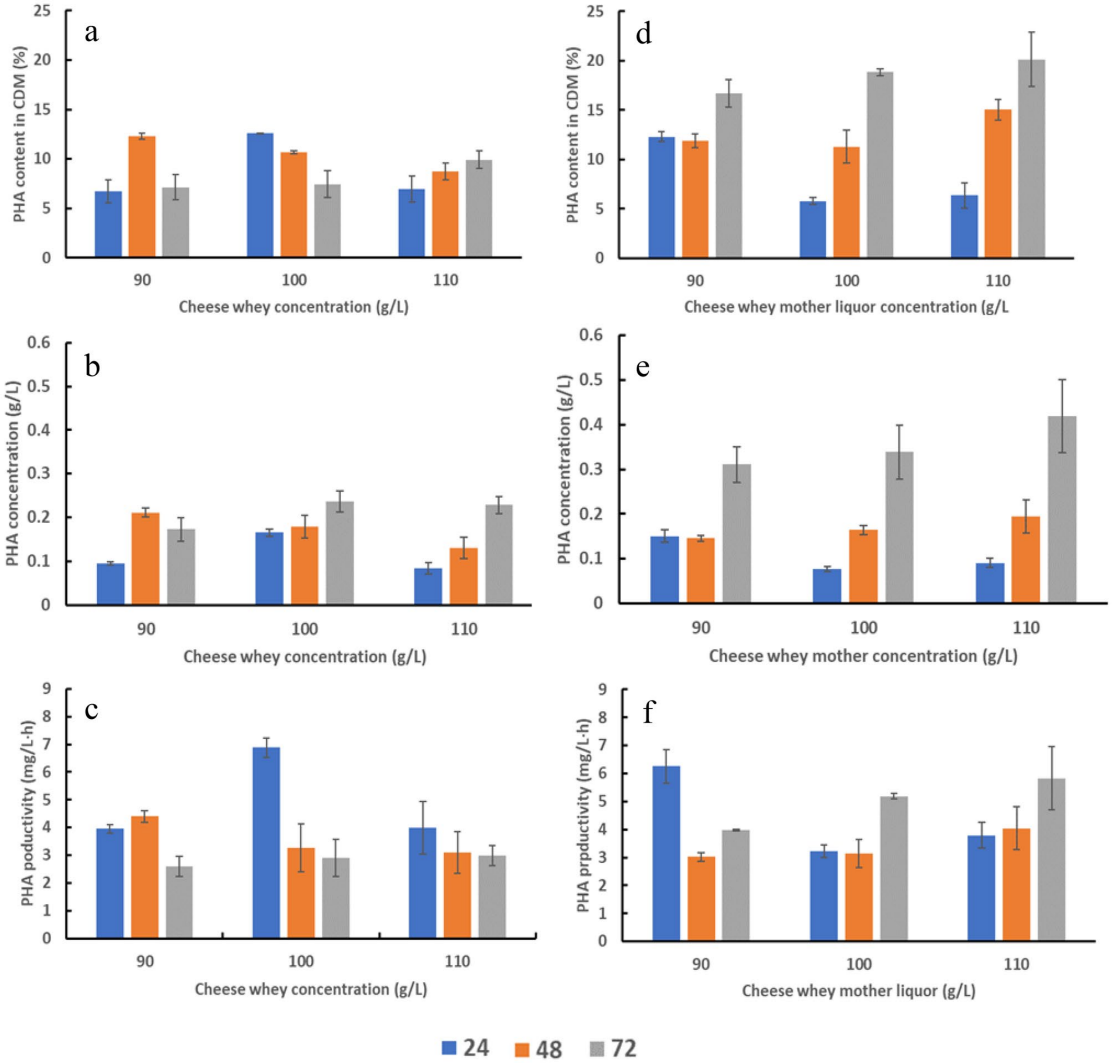
PHA content in CDM, PHA concentration and PHA productivity in 24 h, 48 h and 72 h of the cultivations with (**a**), (**b**), (**c**) cheese whey and (**d**), (**e**), (**f**) cheese whey mother liquor at the concentrations of 90, 100 and 110 mL/L. Mean values are calculated from triplicate measurements.

CW has been previously reported to be challenging for bacterial growth and PHAs production^21^. Our results demonstrated that *H. alkaliantarctica* was capable of utilizing CW and CWML in all tested concentrations for growth as well as for PHA production (Figs. 1a,b; 2a–f). In all cultures the biomass concentration exceeded 1.0 g/L at all measured time-points. These data are in accordance with those reported by Berwig et al.^22^ for *Alcaligenes latus* cultured on milk whey after acid protein precipitation and neutralization with ammonium hydroxide. Lower growth rates (below 0.5 g/L) were determined by Dubey and Mishra^23^ who cultured *H. daqingensis* in a fermentor supplemented with algal biodiesel waste residue. Moreover, our data indicated that CW supported bacterial growth more effectively than CWML (*P* = 0.017). The CDM levels achieved by Możejko-Ciesielska et al.^24^ were higher when *Paracoccus homiensis* was cultivated on CWML compared to CW. Furthermore, our results revealed that the concentration of the substrates used during cultivations of *H. alkaliantarctica* influenced the CDM value. The highest growth rate was reached at 72 h in the cultivation supplemented with CW at the concentration of 100 g/L (Fig. 1a). Moreover, in all experimental variants, the CDM concentration was observed to be higher at 72 h compared to 24 h and 48 h of the cultivations with CW and CWML.

The content of PHAs in *H. alkaliantarctica* was significantly higher when the bacteria were cultured in Bacto Marine Broth supplemented with CWML than with CW (*P* = 0.002) (Fig. 2d). These data are consistent with the results confirmed by Mozejko-Ciesielska et al.^24^ for *Paracoccus homiensis* which converted CWML into PHAs more efficiently than CW. Interestingly, De Andrade et al.^25^ reported the inhibitory effect of CW for PHA storage ability by *Burkholderia sacchari*. The authors observed much lower PHA content (5.1% of CDM) when a mixture of glucose and cheese whey was used as the carbon source compared to glucose (41.4% of CDM). Moreover, our results showed that PHA content and PHA concentration were the highest at 72 h in bacterial cells cultivated in a production medium consisting of 110 g/L CWML (20.1% of CDM and 0.42 g/L, respectively). This PHA content is comparable to the previously reported level produced by *Bacillus flexus* Azu-A2 grown on cheese whey supernatant^26^. A lower PHA level was recorded by Koller et al.^27^ in *Pseudomonas hydrogenovora* cells (12% of CDM) cultured in a 2-L bioreactor with hydrolyzed whey permeate. The enhanced polymer yield was achieved by Pernicova et al.^28^ in the culture of *H. hydrothermalis* supplemented with cheese whey and 3HV precursors such as n-proponol (1.53 g/L of PHA), levulinate (1.63 g/L of PHA) and 1,4-butanediol (1.58 g/L). A higher PHA concentration (2.2 g/L) was observed in *B. megaterium Ti3* cells grown on the mixture of unprocessed dairy waste whey and glucose^29^. Furthermore, the PHA production in *H. alkaliantarctica* cells was inhibited as the CW was added to the culture medium at the concentration of 90 g/L. Although the difference was not statistically significant, in this cultivation, PHAs were synthesized at a lower concentration in 72 h compared to 48 h (*P* > 0.05). Furthermore, our data showed that the biopolyesters were not produced in the Bacto Marine Broth without the supplementation of the analyzed by-products (treated as a control).

As may be observed from the data reported in Fig. 2c, the highest PHA productivity was observed in the cultivation with 100 g/L of CW at 24 h (6.88 mg/L·h). The productivity then decreased at 48 h and 72 h of the cultivations about 48% and 42%, respectively. Lower PHA efficiency was reported by Dubey and Mishra^30^ in the cultivation of *H. hydrothermalis* (MTCC 5445) in a shake flask experiment using 3% dry sea mix instead of fresh water, 3% glycerol and 0.55% peptone. A controlled stress strategy using 1% ethanol enhancing PHA production from cheese whey using *B. megaterium* CCM 2037 reached PHA productivity at the level of 0.03 g/L h^31^. In addition, it was also observed that the mean PHA productivity was significantly lower at 72 h compared to 24 h in all experimental variants with CW (*P* = 0.027). On the contrary, the mean PHA productivity in the cultivations with 100 g/L of CWML was reported to be significantly higher at 72 h than at 24 h (*P* = 0.041). Nevertheless, the PHA productivity levels reported in this study are relatively low as compared to those observed during cultivations in fed-batch bioreactors^27,32^. This is associated with the low growth rate of *H. alkaliantarctica* cultured in Erlenmeyer flasks.

### Effect of dairy industry waste on the monomeric composition of produced PHAs

It is known that high crystallinity, low flexibility and a melting temperature close to the temperature of degradation hamper the potential application of P(3HB) homopolymer^33^. Its market desired properties and postproduction processability can be improved by incorporating the 3HV fraction in the PHA structure. The literature data suggest that microorganisms that were cultured on dairy industry by-products synthesized P(3HB) homopolymer^34^ or P(3HB-co-3HV) copolymer^35^. As can be seen in Table 1, the repeat unit composition of the purified PHAs depended on the type of the by-product and cultivation time. Our results proved that *H. alkaliantarctica* cultured on CWML was able to produce P(3HB) homopolymer in all experimental variants. Furthermore, the analyzed halophile was found to produce P(3HB-co-3HV) copolymer without the addition of any precursors such as valerate, propionate etc. The tendency to incorporate 3HV monomer into the P(3HB) polymer chain was observed only in the cultivations with CW at 48 h and 72 h. A different tendency was observed in the cultivation of *Paracoccus homiensis*, which was able to incorporate 3HV monomers into the P(3HB) polymer chain growing on marine broth medium supplemented with CW and CWML as the only carbon sources^24^. However, Kucera et al.^16^ reported that *H. hydrophila* grown on cheese whey hydrolysate produce P(3HB) homopolymer at 72 h of the cultivation. Moreover, our results suggest that the content of the 3HB and 3HV fractions was related to the substrate concentration and was changeable at different culture times. The 3HV portion in the copolymer reached the highest value when *H. alkaliantarctica* was fed at 100 g/L of CW. In a study on *Pseudomonas hydrogenovora*, the concentration of 3HB and 3HV fractions was found to depend on the amount of enzymatically hydrolyzed whey permeate used as carbon source^36^. This finding is in accordance with the results reported by Mozejko-Ciesielska et al.^24^ who showed that when the PHA production medium was supplemented with 70 mL/L of CW, the 3HV content reached the highest value of 60.59 mol% compared to other substrate concentrations used. Furthermore, in our study, a higher 3HV content was determined at 72 h compared to 48 h for all CW concentrations. The level of 3HV monomer in the chain of produced PHAs was comparable to the previous reported by Pais et al.^35^ who cultured *Haloferax mediterranei* in a medium supplemented with hydrolyzed cheese whey. However, higher 3HV content in P(3HB-co-3HV) copolymer was observed when *Halomonas* sp. SF2003 was cultivated on agro-industrial effluent with valerate as a co-substrate^37^. It was suggested that a high 3HV concentration could be related to the complex composition of carbohydrates in the industrial effluent.

**Table 1:** Monomeric composition of PHAs extracted in 24 h, 48 h and 72 h of the cultivations with cheese whey and cheese whey mother liquor at the concentrations of 90, 100 and 110 mL/L.

	24 h		48 h		72 h	
	3HB (mol%)	3HV (mol%)	3HB (mol%)	3HV (mol%)	3HB (mol%)	3HV (mol%)
Cheese whey (g/L)						
90	100.00	0.00	99.39	0.61	98.94	1.06
100	100.00	0.00	99.50	0.50	98.66	1.34
110	100.00	0.00	98.95	1.05	98.80	1.20
Cheese whey mother liquor (g/L)						
90	100.00	0.00	100.00	0.00	100.00	0.00
100	100.00	0.00	100.00	0.00	100.00	0.00
110	100.00	0.00	100.00	0.00	100.00	0.00

**Figure 3:**
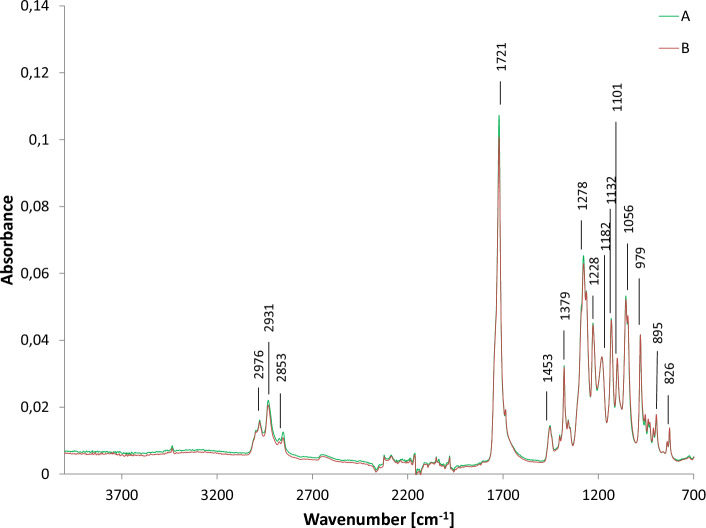
FTIR spectra of P(3HB-co-3HV) extracted from the cultivation with cheese whey (A) and P(3HB) extracted from the cultivation with cheese whey mother liquor (B).

PHA biofilms identified by GC–MS as P(3HB) and P(98.66% HB-co-1.34% HV) were chosen for FTIR analysis (Fig. 3). Several bands were observed in the FTIR spectra, which were assigned to functional groups characteristic for P(3HB) and P(3HB-co-3HV)^38^. The absorption band in the range 3100–2700 cm^−1^ with peaks appearing at 2976, 2931 and 2853 cm^−1^ is associated with the –CH_3_ and –CH_2_ groups. Moreover, it is caused by the symmetrical and asymmetrical C-H stretching bonds present in the above-mentioned groups^39^. A single peak band with a maximum of 1721 cm^−1^ corresponds to the C=O carbonyl groups found in macromolecules. This could be related to an amorphous region of the extracted biopolyester^40^. The intense absorbance at 1722 cm^−1^ was detected in P(3HB) extracted from *Pseudomonas putida* SS9 indicated that the polyesters represent 3-hydroxyalkanoates^34^. In the spectrum range from 1500 to 800 cm^−1^, the so-called fingerprint region, several bands with numerous peaks were recorded. The peaks with maxima at 1453, 1379, 979, 895 and 826 cm^−1^ are associated with the occurrence of symmetrical and asymmetrical scissor C–H bonds indicating –CH_3_ and –CH_2_ groups, as well as C–C bonds. It has been previously shown that a strong vibration at 1379 cm^−1^ is characteristic for PHA^41^. Moreover, a series of absorption bands appearing at 1278, 1228, 1182, 1132, 1101 and 1056 cm^−1^ are caused by the C–O–C bond.

### Influence of dairy industry waste on the material properties of the produced PHAs

**Figure 4:**
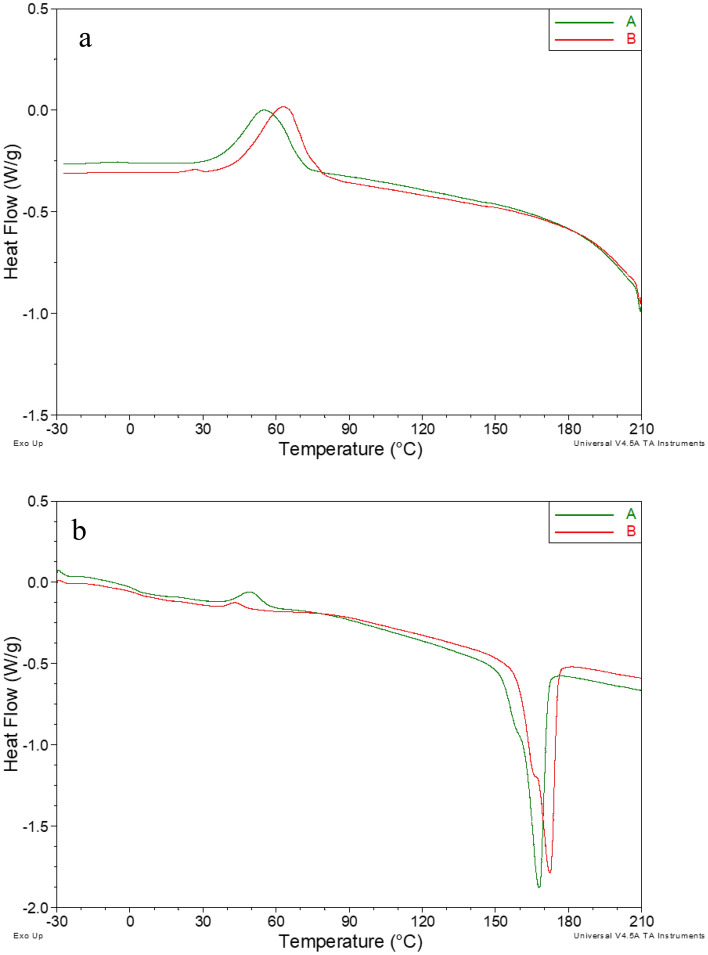
DSC curves (**a**) cooling, (**b**) second heating of tested materials: (A) P(3HB-co-3HV) extracted from the cultivation with cheese whey; (B) P(3HB) extracted from the cultivation with cheese whey mother liquor.

**Figure 5:**
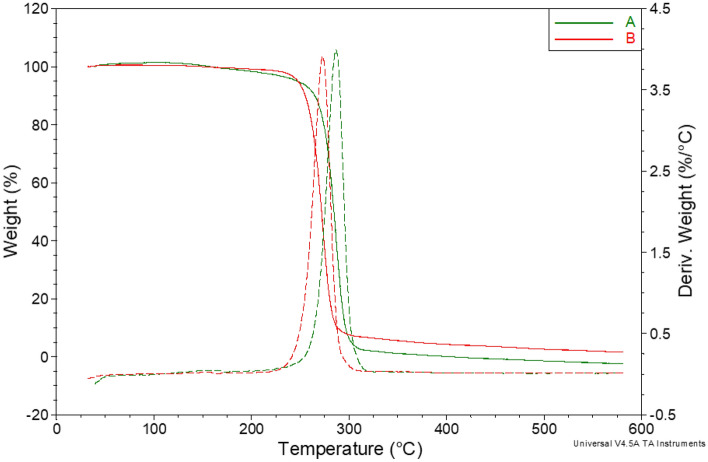
TG (solid line) and DTG (dash line) curves of selected polymers: (A) P(3HB-co-3HV) extracted from the cultivation with cheese whey, (B) P(3HB) extracted from the cultivation with cheese whey mother liquor.

Thermal properties of the PHA biofilms identified by GC–MS as P(3HB) and P(98.66% HB-co-1.34% HV) were examined by DSC and TG analysis (Figs. 4a,b, 5). During cooling on DSC curves of both materials, exothermic crystallization peaks were observed. The temperature of the crystallization (T_c_) of P(3HB) homopolymer was 63.2 °C. The presence of 3HV mer units in the polymer chain of P(3HB-co-3HV) copolymer reduced the T_c_ value to 55.4 °C. Moreover, the intensity of the crystallization process was dependent on the PHA monomeric composition. The value of enthalpy of the crystallization process (ΔH_c_) decreased from 47.8 J/g for the homopolymer to 41.4 J/g for the copolymer. The presence of a 3HV fraction in the PHA structure also caused a decrease in the glass transition temperature (T_g_) determined from the heating curve. T_g_ of P(3HB) was 3.3 °C and dropped to 2.7 °C for P(3HB-co-3HV). Lower T_g_ (1.01 °C) was detected in P(3HB) biofilm produced by *Pseudomonas putida* SS9 cultured on CW^34^. Similar T_g_ value for P(3HB-3HV) was reported by Szacherska et al.^42^ who extracted the copolymer from *Paracoccus homiensis* cells growing on short- and medium chain carboxylic acids derived from acidogenic mixed culture fermentation of acid whey. The influence of the 3HV monomer was also visible in the characteristics of cold crystallization and melting processes. The incorporation of the 3HV fraction in the biopolymer structure increased the temperature of the cold crystallization process (T_cc_) and the intensity of this process was determined based on changes in enthalpy (ΔH_cc_). The same observation was made during the analysis of the PHAs extracted from *Paracoccus homiensis* cells cultured on CW and CWML^24^. Moreover, our results revealed that T_cc_ and ΔH_cc_ values of the extracted P(3HB) reached 42.8 °C and 28.2 J/g, respectively, whereas these parameters were determined to be higher for P(3HB-co-3HV) copolymer 49.8 °C and 34.2 J/g, respectively.

An opposite effect was observed for the melting process. The nature of the process curve was similar for both materials. In both cases, the observed melting peak was bimodal, with a very small arm at lower temperatures. The reason for this phenomenon is probably the earlier melting of the crystalline phase formed in the cold crystallization process, which is usually characterized by lower melting temperatures. However, the content of 3HV units reduced the maximum temperature of the melting process (T_m_) and the position of the second arm, consequently reducing the intensity of this process. A lower T_m_ value was determined for the analyzed copolymer (T_m_ = 167.9 °C) compared to the P(3HB) homopolymer (T_m_ = 172.3 °C). Gahlawat and Soni^43^ found that adding a 3HV monomer to an scl-copolymer extracted from *Cupriavidus necator*, lowers the T_m_ value. Additionally, the degree of crystallinity (X_c_) was differed between the extracted PHAs indicating varying tendencies towards crystallization and cold crystallization. Our data revealed that the X_c_ value of P(98.66% HB-co-1.34% HV) was lower (X_c_ = 35.9%) compared to the extracted homopolymer (X_c_ = 42.9%). Crystallinity is reported to be a parameter that influences in vitro biocompatibility of the PHAs^44^. T_m_ values reported in our study were comparable to the levels of PHAs produced by *H. salina* (T_m_ = 172.3 °C)^45^; *H. nitroreducens* (T_m_ = 170.0 °C)^14^; *H. campisalis* (T_m_ = 170.0 °C)^15^. However, higher melting temperatures (from 178.1 to 180.5 °C) were found for the PHA extracted from *H. halophila* and was dependent on the NaCl concentration in the production medium^16^. The above-mentioned discrepancies in T_m_ levels indicate different values of molecular weight of the produced PHAs^46^.

Furthermore, both tested materials had the same thermal resistance determined from temperature corresponding to 5% weight loss (T_5%_). T_5%_ was observed in the temperature of about 247.0 °C for both analyzed biopolymers. However, the kinetics of this process was different. The incorporation of a 3HV fraction in the polymer chain accelerated the initial stage of the degradation process^47^. T_max_ value was reported to be lower for P(3HB-co-3HV) (273.0 °C) as compared to P(3HB) (286.7 °C) (Fig. 5). Furthermore, our previous study on *Paraccocus homiensis* grown on CW and CWML proved that PHA copolymer extracted from bacterial cells fed with CW had lower thermal resistance than the copolymer from the culture with CWML^24^. Additionally, the temperature at which the copolymer reached 50% weight loss (T_50%_) was lower. The incorporation of 3HV monomer into the PHA structure also influenced the final stage of degradation process by increasing the temperature corresponding to a 95% loss of the biopolymer mass (T_95%_). T_95%_ was reported to be 368.5 °C for the analyzed copolymer and 302.5 °C for the homopolymer. The thermal stability of the produced biopolymers was similar to that revealed by Hassan et al.^48^ who analyzed PHA recovered from *Bacillus* sp. N-2. Lower thermal degradation (240 °C) was observed in P(3HB) produced by *Botryococcus braunii*^49^ and *Halomonas alkalicola M2*^50^.

**Figure 6:**
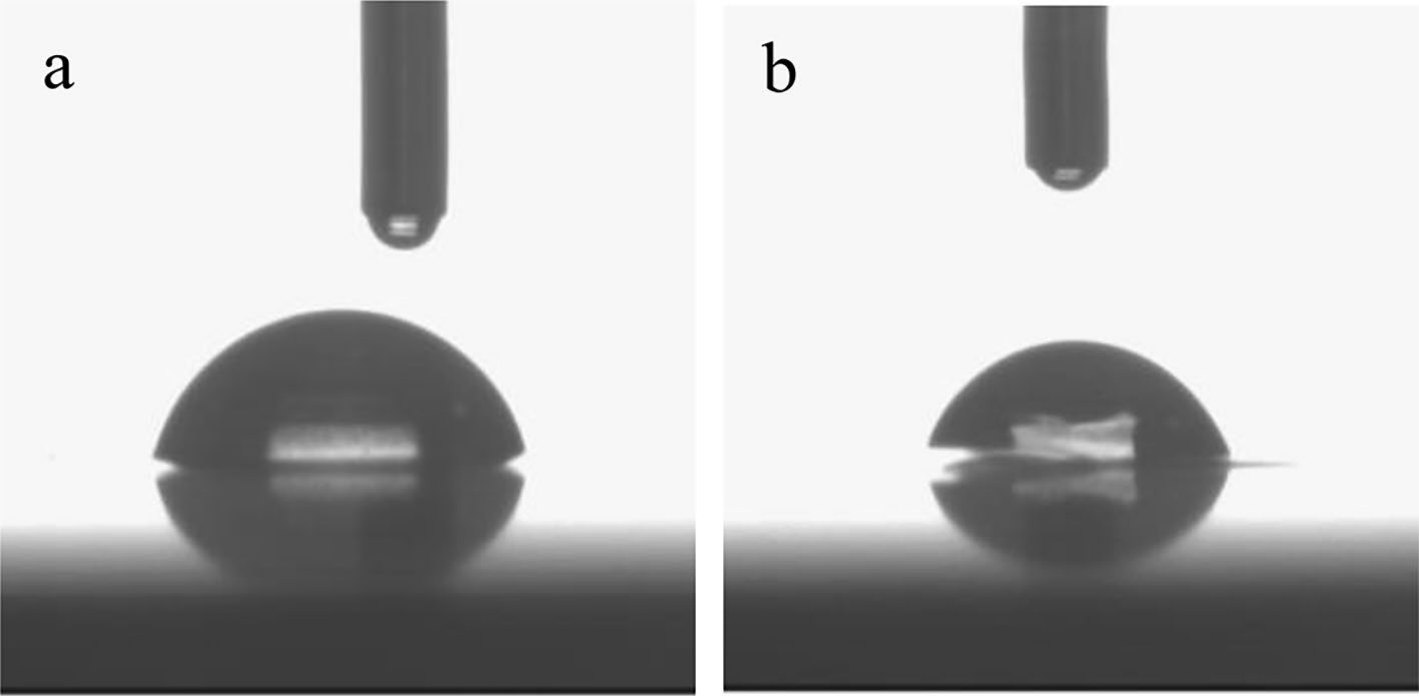
Water contact analysis of: (**a**) P(3HB-co-3HV) extracted from the cultivation with cheese whey, (**b**) P(3HB) extracted from the cultivation with cheese whey mother liquor.

The water contact angle was used to evaluate the hydrophobicity and hydrophilicity characters of PHA samples what play a crucial role in the technological steps involved PHA biofilms. These parameter could be affected by roughness, liquid surface tension or surface free energy^51^. As can be seen in Fig. 6a and b, the analyzed PHA showed similar surface characteristics. Higher water contact angles were recorded for P(3HB) (79.7° ± 2.0) compared to P(3HB-co-3HV) (77.6° ± 2.2). Whereas, the incorporation of the 3HV fraction into P(3HB) had an impact on the surface energy (SE) value. Higher SE was observed for the copolymer (36.4 J/cm^2^) as compared to the homopolymer (35.1 J/cm^2^). The water contact angle values seem to be dependent on the carbon source used in the culture medium. In another study, Zhila et al.^52^ confirmed a higher level of the above mentioned parameter for P(3HB-co-3HV-co-3HHx) synthesized from palm oil compared to P(3HB) synthesized from fructose. Shishatskaya et al.^53^ noted that P(3HB) produced in *Cupriavidus necator* culture with glycerol had lower water contact angles compared to those produced with sugars.

## Conclusions

Our data confirmed that *H. alkaliantarctica* is capable of producing scl-homopolymers and scl-copolymers utilizing cheese whey and cheese whey mother liquor as the only carbon sources. This bacterium is able to efficiently incorporate 3HV mers in the PHA chain to make this technological bioprocess economically feasible and industrially important. Moreover, being a halophile it displays other advantages as a platform for value-added copolymer production due to high salinity tolerance that reduces the risk of contamination during cultivation and decreases the overall costs of this biopolymer production^54^. Therefore, there is a need to focus on further studies in order to optimize the culture parameters in bioreactors.

## Materials and methods

### Microorganism

*H. alkaliantarctica* (DSM 15686) was obtained from the German Collection of Microorganisms and Cell Culture (Braunschweig, Germany). It is a moderately halophilic bacterium isolated from salt in the saline lake Cape Russell in Antarctica.

### Cultivations and culture conditions

For PHA production, the cells of *H. alkaliantarctica* cryo-conserved in the presence of 20% (*w/v*) of glycerol were precultured in Bacto Marine Broth (BMB) supplemented with 8% NaCl (*w/v*) at 28 °C with 200 rpm shaking for 16 h. BMB medium contained (per liter): bacto peptone 5 g, bacto yeast extract 1 g, Fe(III) citrate 0.10 g, NaCl 19.45 g, MgCl_2_ 5.90 g, Na_2_SO_4_ 3.24 g, CaCl_2_ 1.80 g, KCl 0.55 g, NaHCO_3_ 0.16 g, KBr 0.08 g, SrCl_2_ 34.0 mg, H_3_BO_3_ 22.0 mg, Na_2_SiO_3_·5 H_2_O 4.0 mg, NaF 2.40 mg, Na_2_HPO_4_ 8.0 mg. The bacteria were then transferred into a fresh BMB medium. The inoculation size was 5%. All cultivations were conducted in triplicate in 250-mL Erlenmeyer flasks with 100 mL of culture medium. The shake flask cultivations were supplemented with cheese whey (CW) or cheese whey mother liquor (CWML) in varying concentrations from 90 to 110 g/L. They were collected from one of the dairy industries located in northeastern Poland. They were pre-processed as previously reported by Mozejko-Ciesielska et al.^24^. The CW composition was as follows (%): total solid 13.15; protein 2.46; lactose 15.10. CWML consisted of (%): nitrogen 0.16; sodium 0.36; protein 1.1; chloride sodium 0.90; lactic acid 0.47; lactose 12.6; ash 3.75; total solid 18.44; carbohydrates 13.0; fat < 0.05; saturated fatty acids < 0.1; total sugars 12.0. The cultivations for PHA production were performed at 28 °C for 72 h at 200 rpm.

### Biomass determination and PHA extraction

Samples of the culture broth were harvested for analyses at three time-points (24 h, 48 h and 72 h). In order to determine cell dry mass concentration (CDM), triplicate samples of bacterial cells were centrifuged (11,200×*g* for 10 min). After removing the supernatant, the bacterial pellet was washed with deionized water. The cell pellet was separated from the supernatant using centrifugation (11,200×*g* for 10 min) and then lyophilized for 24 h by Lyovac GT2 System (SRK Systemtechnik GmbH, Riedstadt, Germany). PHA extraction was conducted by shaking the freeze-dried cells in chloroform (purity ≥ 99.8%; Sigma-Aldrich; USA) for five hours at 50 °C. The obtained mixture was further filtered through No. 1 Whatman filter paper and washed with cold 70% methanol. The collected pellet was allowed to evaporate at room temperature. PHA content (% of CDM) was determined in the lyophilized biomass and was defined as the percentage of the ratio of PHA concentration to CDM concentration.

### Analysis of PHAs

PHA quality was determined by Gas Chromatography coupled with Mass Spectrometry (GC–MS QP2010 PLUS, Shimadzu, Japan), as reported by Możejko-Ciesielska and Pokój^55^. The dried biomass was treated by acidic methanolysis. Obtained methyl esters were injected into a BPX70 (25 m × 0.22 mm × 0.25 mm) capillary column (SGE Analytical Science, Victoria, Australia). Helium was used as a carrier gas at 1.38 mL/min. The initial temperature was programmed as 80 °C, followed by an increase to 240 °C at 10 °C/min. The temperature of the ion source was maintained at 240 °C. The quantitative analysis was used P(3HB-co-3HV) copolymer (Sigma Aldrich, USA) as a standard.

Infrared spectra of the PHAs were obtained using a Fourier Transform Infrared Spectrophotometer (FTIR) Nicolet iS10 (ThermoScientific, USA) by attenuated total reflectance mode (ATR-FTIR), in the spectral range from 4000 to 650 cm^−1^^42^.

Thermal analyses were conducted according to the procedures described previously in the literature^56^. Differential scanning calorimetry (DSC) analyses were performed using a Q200 apparatus (TA Instruments, USA). PHA biofilms were scanned at the range from − 30 °C to 210 °C. The glass transition temperature (T_g_), crystallization temperature (T_c_) and enthalpy of the crystallization process (ΔH_c_) were taken from the cooling curve. The biopolymer samples were then heated to 210 °C at a heating rate of 10 °C/min. The glass transition temperature (T_g_), melting point (T_m_), the enthalpy of melting process (ΔH_cc_) and degree of crystallinity (X_c_) were evaluated from the obtained DSC curve^57^.

Thermogravimetric analysis (TGA) was performed using a Q500 apparatus (TA Instruments, USA). The test was conducted over a temperature range from 30 to 600 °C at 10 ºC/min as heating rate. From the thermogravimetric curve, loss temperatures of 5%, 50% and 95% of initial mass were determined as T_5%_, T_50%_ and T_95%_ respectively.

The surface wettability of the PHA films were performed using a DSA 100 goniometer (Krüss GmbH, Germany). In brief, a drop of water was placed on the surface of a test sample, its volume was constantly increased and at the same time the dynamic contact angle was measured. The average value was recorded from ten measurements and surface energy (SE) was then calculated using the Neumann method^58,59^.

### Statistical analysis

All samples were analyzed in triplicate. Statistical evaluation of all data was conducted using STATISTICA v.13.1 (StatSoft, Inc, Tulsa, OK, USA). The Mann–Whitney *U*-test was used to determine the significance (*P* < 0.05) of differences in PHA yield and bacterial cell concentration between experimental variants.

## Data Availability

All data generated or analyzed during this study are present in the paper.
